# Comparative Analysis of Robust Entanglement Generation in Engineered XX Spin Chains

**DOI:** 10.3390/e27070764

**Published:** 2025-07-18

**Authors:** Eduardo K. Soares, Gentil D. de Moraes Neto, Fabiano M. Andrade

**Affiliations:** 1 Programa de Pós-Graduação em Ciências/Física, Universidade Estadual de Ponta Grossa, Ponta Grossa 84030-900, PR, Brazil; 2 College of Physics and Engineering, Qufu Normal University, Qufu 273165, China; 3 Departamento de Matemática e Estatística, Universidade Estadual de Ponta Grossa, Ponta Grossa 84030-900, PR, Brazil; 4 Departamento de Física, Universidade Federal do Paraná, Curitiba 81531-980, PR, Brazil

**Keywords:** quantum spin chains, entanglement generation, XX model, quantum information transfer

## Abstract

We present a numerical investigation comparing two entanglement generation protocols in finite XX spin chains with varying spin magnitudes (s=1/2,1,3/2). Protocol 1 (P1) relies on staggered couplings to steer correlations toward the ends of the chain. At the same time, Protocol 2 (P2) adopts a dual-port architecture that uses optimized boundary fields to mediate virtual excitations between terminal spins. Our results show that P2 consistently outperforms P1 in all spin values, generating higher-fidelity entanglement in shorter timescales when evaluated under the same system parameters. Furthermore, P2 exhibits superior robustness under realistic imperfections, including diagonal and off-diagonal disorder, as well as dephasing noise. To further assess the resilience of both protocols in experimentally relevant settings, we employ the pseudomode formalism to characterize the impact of non-Markovian noise on the entanglement dynamics. Our analysis reveals that the dual-port mechanism (P2) remains effective even when memory effects are present, as it reduces the excitation of bulk modes that would otherwise enhance environment-induced backflow. Together, the scalability, efficiency, and noise resilience of the dual-port approach position it as a promising framework for entanglement distribution in solid-state quantum information platforms.

## 1. Introduction

The advancement of quantum technologies depends crucially on the ability to generate and control quantum resources in a reliable and scalable way [1,2]. Among these resources, quantum entanglement stands out as a fundamental component, enabling powerful protocols to operate within the limits of local operations and classical communication (LOCC) [2]. Entangled states are at the heart of key quantum information processing tasks, such as teleportation [3], sensing [4], and communication [5,6]. In particular, maximally entangled states, such as Bell pairs (EPR states), are essential building blocks for these applications [7].

Although significant advances have been made in generating entanglement with photonic systems [8,9], scalable solid-state quantum processors require architectures that can produce and distribute entanglement on demand, integrated directly with quantum registers [10]. Spin chains have thus emerged as promising candidates for short-range quantum communication and entanglement distribution [11,12,13], due to their tunability and compatibility with solid-state platforms. Physical implementations span electron spins in quantum dots [14], magnetic molecules [15], and endohedral fullerenes [16], making spin chains attractive as modular elements for scalable quantum devices.

There is an extensive body of work exploring spin chains for quantum state transfer [11,17,18], entanglement routing [19,20,21], and quantum bus architectures [22,23,24,25,26,27,28]. However, much of this research focuses on idealized spin-1/2 models and often overlooks practical challenges such as disorder and decoherence. Additionally, the performance of higher-spin chains and comparisons between different entanglement generation schemes remain largely unexplored [29,30,31].

We address these gaps by comparing two entanglement generation protocols based on XY-type spin chains: Protocol 1 (P1), where alternating weak and strong couplings guide quantum correlations toward the chain edges [23,25,32,33,34], and Protocol 2 (P2), which employs symmetric perturbative couplings at both ends to enhance the transport speed and facilitate the buildup of quantum correlations [35,36,37,38]. Using the XX spin model, we systematically investigate the entanglement dynamics for spins s=1/2, 1, and 3/2 under both ideal and noisy conditions. Our analysis is primarily based on extensive numerical simulations. We quantify entanglement using the negativity measure, explicitly including the effects of static disorder, both diagonal and off-diagonal, as well as local decoherence channels within the model. Non-Markovian effects, originating from structured environments with finite memory times or strong system–bath correlations, can significantly influence quantum coherence, entanglement dynamics, and the fidelity of quantum operations [39,40]. Accurately capturing these effects is essential in understanding realistic quantum devices.

To this end, we employ the pseudomode formalism [41,42], a conceptually transparent and computationally efficient approach for the modeling of non-Markovian dynamics induced by reservoirs with Lorentzian or near-Lorentzian spectral densities. In this framework, the environment is effectively replaced by a set of auxiliary damped harmonic oscillators (pseudomodes) that interact directly with the system. This mapping enables the simulation of non-Markovian effects using standard Lindblad master equations, thereby retaining essential memory effects while remaining compatible with widely used numerical techniques.

Although effective Hamiltonians are derived to elucidate the underlying physical mechanisms, our main conclusions are based entirely on the numerical data. Importantly, we observe that the dual-port databus protocol (P2) enables faster and more robust entanglement generation compared to the staggered (P1) scheme, especially in the presence of environmental noise and fabrication imperfections. To demonstrate practical applicability, we benchmark the entangling times and parameter regimes against those accessible in the current solid-state platforms. Our results are relevant to a variety of systems—including trapped ions [43], superconducting qubit arrays [44], nitrogen-vacancy centers in diamond [45], and quantum dot devices [46]—where engineered spin–spin interactions and coherent control have been experimentally realized. This work thus paves the way for deterministic and scalable entanglement sources in next-generation quantum processors and networks [47,48,49,50].

## 2. Model and Methods

**Figure 1:**
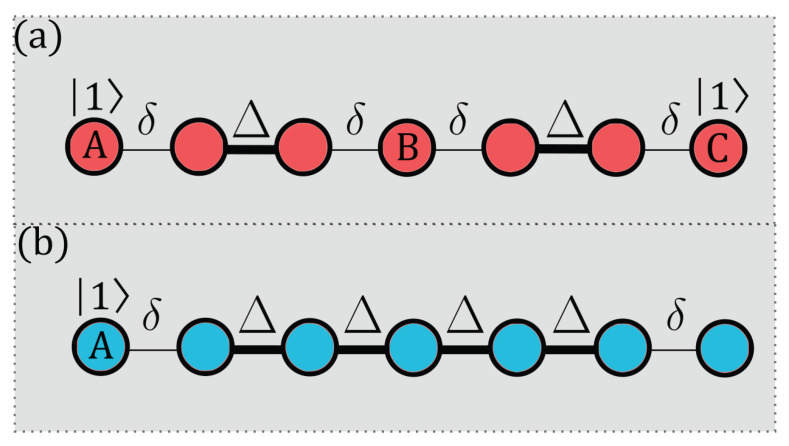
(**a**) P1 and (**b**) P2 architectures. Bold lines represent Δ couplings, while thin lines indicate δ couplings.

We model the system as an *N*-site XX spin chain with Hamiltonian

(1) $$H=\sum_{i=1}^{N-1}J_{i}\left(S_{i}^{x}S_{i+1}^{x}+S_{i}^{y}S_{i+1}^{y}\right)+\sum_{i=1}^{N}B_{i}S_{i}^{z},$$

where $S_{i}^{\alpha }$ denotes spin-*s* operators and $B_{i}$ denotes local magnetic fields. The coupling constant $J_{i}$ alternates between two values, Δ and δ, depending on the position in the chain (see Figure 1).

The system evolves under the Lindblad master equation [51,52,53], which describes the combined unitary and dissipative dynamics:

(2) $$\dot{\rho }=-i\left[H,\rho \right]+\gamma \sum_{i=1}^{N}\left(S_{i}^{z}\rho S_{i}^{z}-\frac{1}{2}S_{i}^{z}S_{i}^{z},\rho \right).$$

The dissipative term proportional to γ introduces local pure dephasing, a common and critical source of decoherence in quantum systems. This type of noise models the loss of quantum coherence without energy exchange with the environment and is particularly relevant in platforms such as superconducting qubits [44,54,55,56], trapped ions [57], and ultracold atom simulators of spin chains [58,59]. In such systems, fluctuations in the local environment or control parameters often lead to dephasing noise that dominates other dissipative processes. Although we set γ=0 unless otherwise stated to focus on the coherent dynamics, we also consider finite γ to assess the robustness of quantum correlations under realistic conditions.

We assess entanglement between the chain ends via the negativity [60,61,62], defined as

(3) $$N\left(\hat{\rho }\right)=\frac{{∥{\hat{\rho }}^{T_{A}}∥}_{1}-1}{2},$$

where ${\hat{\rho }}^{T_{A}}$ is the partial transpose of the quantum state $\hat{\rho }$ with respect to subsystem A, and ${∥\hat{Y}∥}_{1}=\left|\hat{Y}\right|=\sqrt{{\hat{Y}}^{†}\hat{Y}}$ denotes the trace norm or the sum of the singular values of the operator $\hat{Y}$. Alternatively, negativity can be calculated as $N\left(\hat{\rho }\right)=\sum_{i}\left(\right|\varepsilon_{i}\left|-\varepsilon_{i}\right)/2$, where $\varepsilon_{i}$ denotes the eigenvalues of the partially transposed density matrix $\hat{\rho }$. The maximum attainable value of $N(\hat{\rho })$ is constrained by the dimensionality of the Hilbert space, which depends on the spin magnitude *s*. Because we work across different dimensionality systems, we normalize our negativity calculations relative to the theoretical maximum for the specific spin value *s*, corresponding to the negativity of a maximally entangled state in the relevant Hilbert space.

To assess whether the protocols generate a Bell state when the maximal possible negativity N is achieved, we compute the fidelity [63]

(4) $$\begin{array}{c} F\left(\hat{\rho },\hat{\sigma }\right)=\left(\mathrm{tr}\sqrt{\sqrt{\hat{\rho }}\;\hat{\sigma }\;\sqrt{\hat{\rho }}}\right) \end{array}$$

where $\hat{\rho }$ is the density matrix of the generated state, and $\hat{\sigma }$ is the density matrix associated with the target state. Fidelity of F=1 indicates the perfect preparation of the target state, while values below unity quantify deviations from this.

### Entanglement Generation Protocols

We investigate two different entanglement generation protocols, illustrated in Figure 1, both designed to mediate long-range entanglement between boundary spins in a finite chain. Unlike traditional quantum communication setups focused on state transfer fidelity, our objective here is the efficient and robust creation of quantum correlations—specifically entanglement—between distant parties.

P1 employs a staggered spin chain initialized in the state

(5) $$|\psi (0)\rangle =|1\rangle_{A}⊗|0\rangle^{⊗N-2}⊗|1\rangle_{C}.$$

Moreover, it evolves unitarily under the system Hamiltonian with $B_{i}=0$. In this configuration, the boundary spins *A* and *C* are initially excited, while the intermediate sites remain unexcited. The entanglement dynamics, in this case, arise from coherent exchange interactions distributed across the entire chain.

For P2, the chain is initialized in the state

(6) $$|\psi (0)\rangle =|1\rangle_{A}⊗|0\rangle^{⊗N-1},$$

but, here, a single excitation is localized at the sender (s) site, while all other spins, including the receiver (r) at the opposite end, begin in the unexcited state. For a spin-*s* system, we define the computational basis states in terms of the eigenstates of the $S_{z}$ operator. Specifically, for spin-1, the basis states are |0⟩≡|m=−1⟩, |1⟩≡|m=0⟩, and |2⟩≡|m=+1⟩. For spin-3/2, the definitions are |0⟩≡|m=−3/2⟩, |1⟩≡|m=−1/2⟩, |2⟩≡|m=+1/2⟩, and |3⟩≡|m=+3/2⟩.

Initial state preparation follows a consistent scheme across the different spin systems. In P1, the bulk spins are initialized in the minimal $S_{z}$ eigenstate, |m=−s⟩, corresponding to the lowest indexed basis state |0⟩, while the boundary spins are set to the maximal eigenstate, |m=+s⟩, corresponding to the highest indexed basis state. In contrast, P2 initializes all spins uniformly in the minimal eigenstate |m=−s⟩. A zero magnetic field $B_{i}=0$ is applied in the bulk, while carefully engineered, *optimized boundary magnetic fields* are applied at the extremities to enhance the coherent buildup of long-range entanglement.

A central advantage of P2 is that the bulk (spins 2 through N−1) remains largely unexcited during evolution. That is, the intermediate spins undergo only *virtual excitation*, which avoids a significant population of the bulk and enables the boundary spins to interact effectively as if they were directly coupled. This virtual coupling mechanism reduces the influence of imperfections within the chain, such as diagonal and off-diagonal disorder or local dephasing, thereby supporting the robust generation of entanglement between the sender and receiver.

Although structurally reminiscent of state transfer protocols, the goal here is not to maximize the transfer fidelity but to exploit coherent dynamics for the fast and resilient generation of entanglement. This distinction is central to our investigation, and we provide detailed numerical results in the following section to validate the effectiveness of both protocols under various conditions. In particular, we systematically compare their performance across different spin magnitudes, analyze their resilience to disorder and decoherence, and explore how the introduction of site-dependent magnetic fields ($B_{i}\neq 0$) affects entanglement generation.

The analysis reveals that P2 offers three key advantages over P1: (i) it achieves maximal entanglement between boundary spins on shorter timescales, with this effect being particularly pronounced in the spin-1/2 case; (ii) it demonstrates enhanced robustness against both diagonal and off-diagonal disorder, as well as dephasing noise; and (iii) it maintains high entanglement generation efficiency even in higher-spin systems (s=1 and 3/2), where P1 shows reduced effectiveness.

These benefits stem from the engineered boundary control and the architecture’s ability to harness virtual excitations for indirect but coherent boundary coupling, effectively bypassing the detrimental effects of bulk-mediated decoherence. The validity of these claims and the quantitative characterization of these mechanisms will be fully explored in the next section. We note that various mechanisms have been explored in the literature for the generation of long-distance entanglement in spin chains, providing complementary approaches to the coherent transfer-based protocols that we analyze. Static methods, such as engineered couplings in dimerized chains and entanglement routers [24,64], can create ground states with naturally entangled boundaries. Dissipative approaches [65,66,67] utilize controlled environmental coupling to prepare entangled steady states. More recent developments include measurement-based post-selection [68] and the exploitation of topologically protected edge modes [69]. Additionally, numerous protocols for high-fidelity quantum information transfer [28] could potentially be adapted to maximize boundary entanglement. While we do not provide a quantitative comparison here, these diverse methods highlight the rich landscape of available techniques and position our protocols within this broader research context.

## 3. Results

### 3.1. Benchmark Without Noise: Dynamics in Pristine Chains

**Figure 2:**
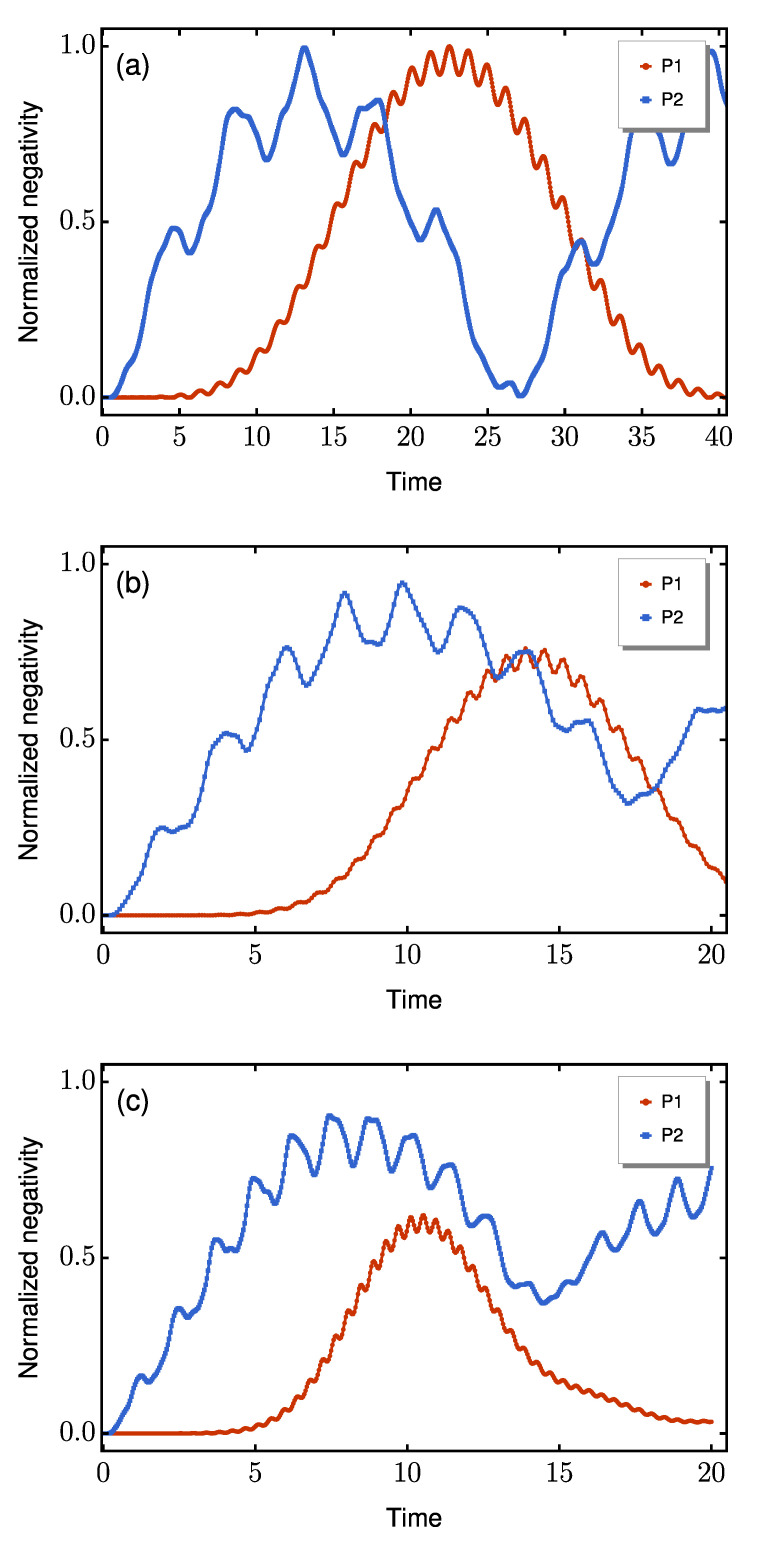
Time evolution of the end-to-end negativity for (**a**) s=1/2, (**b**) s=1, and (**c**) s=3/2. All traces correspond to the same dimerization ratio Δ/δ=10. Results are shown for P1 (red curves) and P2 (blue curves).

To establish a baseline, we first compare the coherent dynamics of the two architectures in the absence of disorder or decoherence for N=7. Figure 2 shows the time evolution of the end-to-end negativity for spin magnitudes s=1/2, 1, and 3/2. The results demonstrate that P2 reaches its first entanglement maximum significantly more quickly than P1 while maintaining robust performance across different spin dimensions. The quantitative data extracted from these curves are presented in Table 1.

**Figure 3:**
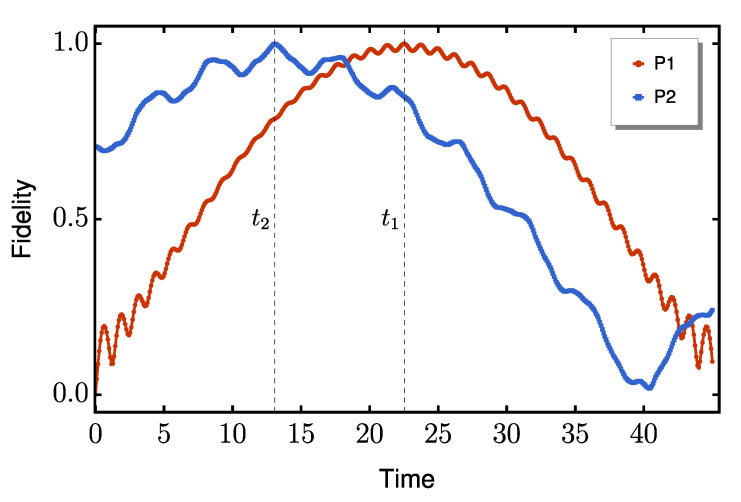
Time evolution of fidelity when considering $\left|\psi^{+}\right\rangle$ as the target state. The red line represents P1, while the blue line represents P2. The time at which maximal entanglement is achieved for each protocol is marked with a dashed vertical line. We set the dimerization ratio to Δ/δ=10.

In all cases, P2 exhibits faster entanglement generation and consistently achieves higher negativity values. In particular, for s=1/2, both protocols output the Bell state $|\psi^{+}\rangle =\left(|01\rangle +|10\rangle \right)/\sqrt{2}$ when maximally entangled, although P2 requires a local −π/2 rotation to be applied on the *z*-axis of the qubit at site *N*, via the application of the $R_{z}$ gate. The fidelity dynamics concerning the target state $\left|\psi^{+}\right\rangle$ are shown in Figure 3, where the necessary rotation for P2 has already been applied before the fidelity calculation.

**Figure 4:**
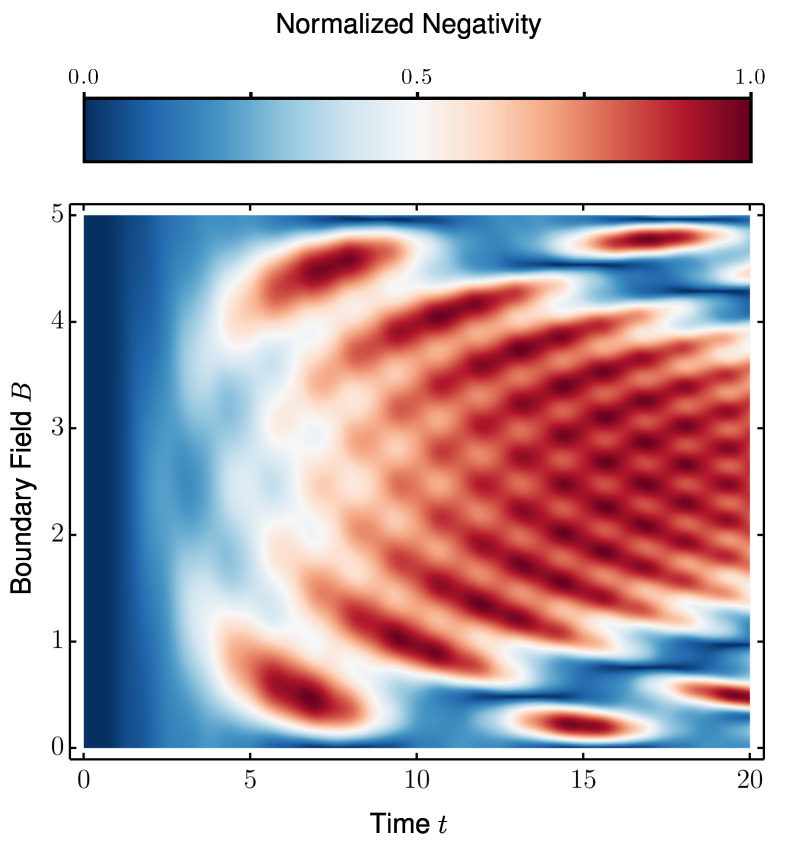
Contour plot of negativity values as a function of time and the magnetic field applied to the boundaries for a N=7 chain. We set the dimerization ratio to Δ/δ=10.

P2 can also be extended to arbitrary chain lengths *N* when s=1/2, making it possible to obtain maximally entangled states for higher values of *N*. For each system size, we only need to optimize the boundary magnetic field *B* to find the maximal possible negativity between terminal spins. This simple adjustment of *B* for different *N* consistently yields maximal or near-maximal entanglement, demonstrating the scalability of the protocol. To determine the optimal value of *B*, we numerically analyze the relationship between *B* and the resulting negativity, identifying the parameter regimes and time scales that maximize the negativity, as shown in Figure 4.

### 3.2. Robustness of the Spin-1/2 Protocol

The performance advantage of P2 is most relevant when it survives realistic imperfections. Therefore, we investigate its stability against static disorder, focusing on the spin-1/2 chain as a representative and experimentally accessible platform. To achieve this, we quantitatively assess its stability by introducing static disorder in spin-1/2 chains—a platform chosen for both its theoretical tractability and its experimental relevance. The analysis focuses on two fundamental channels of disorder that reflect distinct physical origins.

First, *on-site (diagonal) disorder* tests P2’s sensitivity to variations in the fine-tuned boundary magnetic fields essential for its operation. To model fabrication-induced energy offsets, we introduce random local fields $h_{i}=Ed_{i}\delta$ with $d_{i}\in \left[-0.5,0.5\right]$ uniformly distributed, where *E* scales the disorder strength relative to weak coupling δ. This modifies the Hamiltonian as

(7) $$H\to H+E\delta (d_{1}S_{1}^{z}+d_{N-1}S_{N-1}^{z}).$$

For comparison, we apply identical perturbations to P1, establishing a performance baseline under equivalent conditions.

Second, *coupling (off-diagonal) disorder* captures imperfections in exchange interactions arising from material defects or control errors. The modified couplings $J_{i}\to J_{i}+Ed_{i}\delta$ yield the adjusted Hamiltonian

(8) $$H\to \sum_{i=1}^{N-1}\left(J_{i}+Ed_{i}\delta \right)\left(S_{i}^{x}S_{i+1}^{x}+S_{i}^{y}S_{i+1}^{y}\right).$$

We investigate three distinct regimes: (i) pure on-site disorder, (ii) pure coupling disorder, and (iii) simultaneous disorder. For each disorder strength *E*, we ensure statistical reliability by computing the end-to-end peak negativity across $10^{3}$ realizations. Specifically, for each realization, we simulate the time evolution under the corresponding disordered Hamiltonian, record the maximum entanglement value attained during the evolution, and then average this value over all realizations. The resulting data points, plotted for varying *E*, quantify the robustness of both protocols against realistic experimental imperfections.

**Figure 5:**
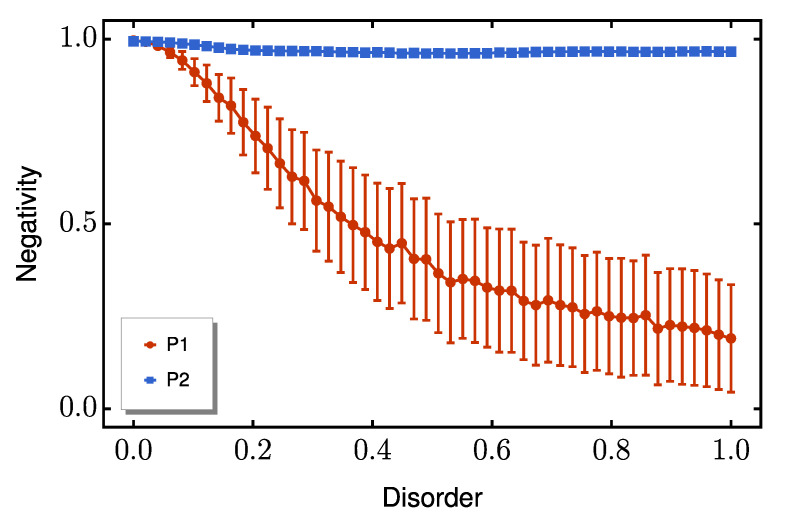
Average peak negativity as function of diagonal disorder strength *E*. The red line represents P1, while P2 is shown as a blue line. The red and blue bars indicate the standard deviation from the mean for each protocol. We set the dimerization ratio to Δ/δ=10.

Three cases are analyzed: (i) pure on-site disorder, (ii) pure coupling disorder, and (iii) both disorder types acting simultaneously. We begin by analyzing pure on-site disorder. Figure 5 shows that P2 maintains excellent performance even at high disorder strengths, while P1 suffers significant entanglement degradation. This robustness is particularly valuable for practical implementations, as it allows for high entanglement generation (high negativity values) despite imperfections in the applied boundary magnetic fields.

**Figure 6:**
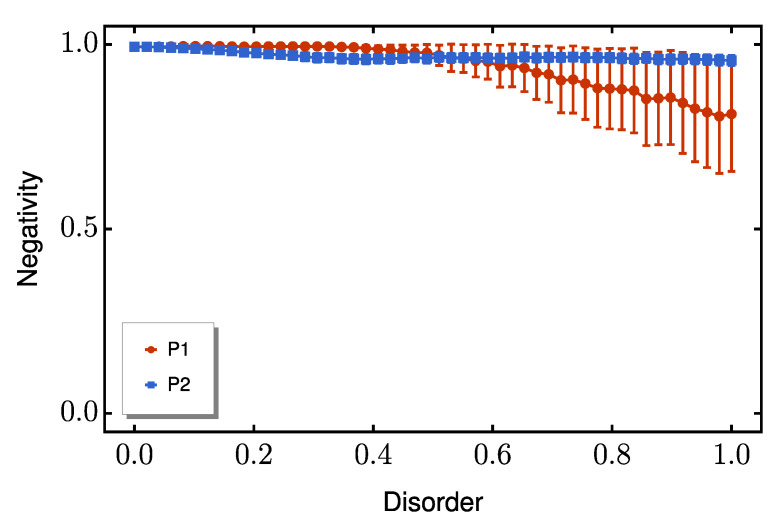
Average peak negativity as function of off-diagonal disorder strength *E*. The red line represents P1, while P2 is shown as a blue line. The red and blue bars indicate the standard deviation from the mean for each protocol. We set the dimerization ratio to Δ/δ=10.

**Figure 7:**
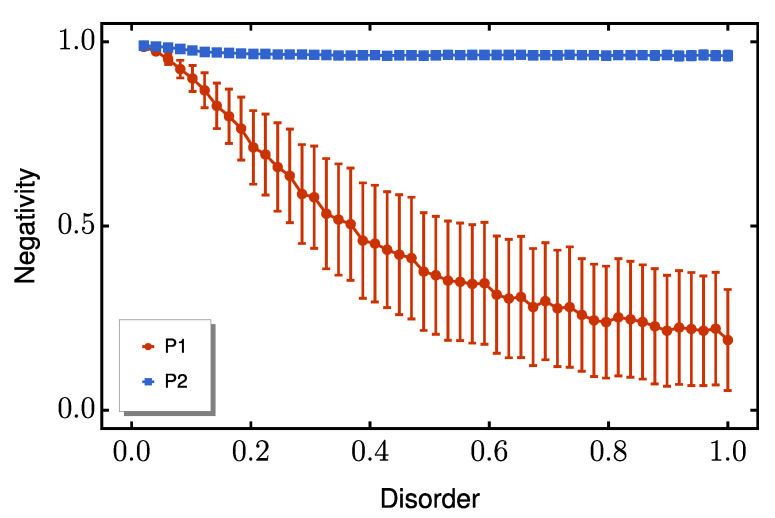
Average peak negativity as function of combined diagonal and off-diagonal disorder strength *E*. The red line represents P1, while P2 is shown as a blue line. The red and blue bars indicate the standard deviation from the mean for each protocol. We set the dimerization ratio to Δ/δ=10.

A similar advantage emerges for coupling disorder (Figure 6), where P2 maintains substantial entanglement (N≈0.8) even at values of E≈0.75δ. Interestingly, when diagonal and off-diagonal disorder are present simultaneously, P2 continues to outperform P1, as shown in Figure 7. This consistent superiority across all disorder regimes confirms P2’s exceptional resilience to typical solid-state fabrication imperfections.

### 3.3. Resistance to Dephasing

**Figure 8:**
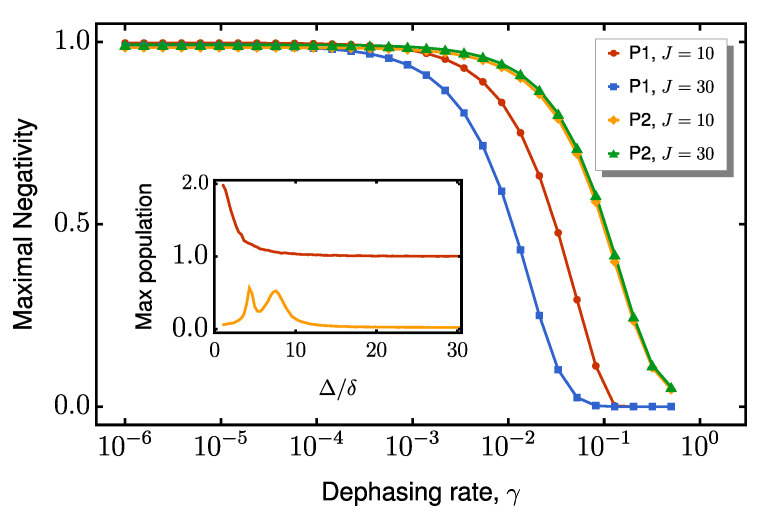
Peak end-to-end negativity as function of boundary dephasing rate γ, shown for two coupling regimes: Δ=10 and Δ=30, with δ=1. The main plot compares the performance of protocols P1 and P2, highlighting the enhanced robustness of P2, which exhibits a slower decay in entanglement under increasing dephasing. The inset displays the maximum population in the bulk channel for each protocol, demonstrating that P2 maintains significantly lower excitation in the intermediate spins across both coupling regimes.

A ubiquitous source of decoherence in solid-state devices is the pure dephasing of the qubits that terminate the spin chain and interface it with external control circuitry. To assess its influence, we numerically evolve the *full* Lindblad master equation [see Equation (2)], in which the term

(9) $$\gamma \sum_{i=1}^{N}\;\left(S_{i}^{z}\hat{\rho }S_{i}^{z}-\frac{1}{2}\left\{{\left(S_{i}^{z}\right)}^{2},\hat{\rho }\right\}\right),$$

already models local dephasing for every site. By sweeping the dephasing rate γ and recording the peak end-to-end negativity, we obtain the curves in Figure 8. The qualitative difference in the decay trends between protocols P1 and P2 can be understood through the lens of the effective Hamiltonian derived in Appendix A.

In the dispersive regime (δ≪Δ), and following well-established perturbative techniques [70,71], the spin chain dynamics underlying P2 can be effectively reduced to a two-qubit model,

(10) $$H_{\mathrm{eff}}=\chi \left(S_{e}^{+}S_{r}^{-}+S_{e}^{-}S_{r}^{+}\right),\;\chi =\sum_{k}\frac{{\bar{\lambda }}_{k}^{2}}{\zeta_{k}},$$

with an accompanying Lindblad term

(11) $${\dot{\rho }}_{\mathrm{eff}}=\frac{\Gamma }{2}\;\sum_{j=e,r}\;\left(2S_{j}^{z}\rho_{\mathrm{eff}}S_{j}^{z}-\left\{{\left(S_{j}^{z}\right)}^{2},\rho_{\mathrm{eff}}\right\}\right),$$

and $\lambda_{k}$ defined as in Equation (A5). Here, the dephasing rate Γ is renormalized as

(12) $$\Gamma =\gamma \sum_{k}\frac{{\bar{\lambda }}_{k}^{2}}{\zeta_{k}^{2}}\propto \gamma {\left(\frac{\lambda }{J}\right)}^{2},$$

indicating that dephasing within the bulk enters only at higher order. Since the chain spins remain virtually unexcited, the entangled state decays predominantly through the suppressed rate Γ, explaining the gradual decline in negativity in P2 (Figure 8). In contrast, P1 lacks this protection, leading to more pronounced sensitivity to dephasing.

This distinction is clearly illustrated in the inset of Figure 8, which plots the maximum bulk excitation as a function of the coupling ratio Δ/δ for both protocols. P2’s enhanced resilience to dephasing arises from its effective decoupling from the bulk, whereas P1 relies on direct excitation transport through the chain, making it significantly more vulnerable to noise. The key difference lies in how entanglement is generated: P1 requires the physical propagation of excitations through intermediate spins before boundary entanglement can be established. As each of these intermediate spins becomes populated, the system accumulates dephasing noise at each site. This sequential exposure results in a larger bulk population (see the red curve in the inset of Figure 8) and sharper degradation in entanglement. In contrast, P2 consistently maintains low bulk occupation (orange curve in the inset of Figure 8) across all tested Δ/δ ratios. This population suppression directly explains the significantly flatter negativity decay curve for P2 in the main panel of Figure 8: by avoiding the buildup of noise along the chain, it preserves the entanglement more effectively under dephasing.

The enhanced protection in P2 comes from two key factors related to the dimerization ratio Δ/δ. First, higher values push the system deeper into the dispersive regime, suppressing the real chain occupation. Second, the effective dephasing rate $\Gamma \propto \gamma {\left(\lambda /J\right)}^{2}$ decreases quadratically with $\zeta_{k}$, explaining why P2’s negativity curves in Figure 8 decay increasingly slowly as Δ/δ grows. The combination of these effects, i.e., the minimal bulk population and suppressed Γ, gives P2 its characteristically flat negativity decay.

In contrast, P1’s excitation-mediated transport remains fundamentally exposed to dephasing regardless of Δ/δ, as its physical propagation mechanism inevitably populates intermediate sites. While stronger dimerization may slightly reduce bulk occupation, it cannot eliminate the accumulation of sequential noise along the chain. This stark difference highlights the central advantage of virtual tunneling: by avoiding real excitations in the bulk, P2 naturally decouples from noise sources while maintaining efficient end-to-end entanglement generation.

### 3.4. Non-Markovian Effects

The accurate description of open quantum systems is essential for the development and operation of noisy intermediate-scale quantum (NISQ) devices [72]. In these devices, the interaction with the environment often leads to decoherence and dissipation that cannot be fully captured by memoryless (Markovian) approximations. Non-Markovian effects, which arise due to structured environments with significant memory times or strong system–bath correlations, can substantially influence quantum coherence, the entanglement dynamics, and ultimately the fidelity of quantum operations [39,40]. For example, in superconducting qubits, non-Markovian noise from two-level system defects leads to coherence revivals [73], while, in quantum dots, phonon-induced memory effects cause a non-exponential decay in entanglement [74].

Several theoretical and numerical approaches have been developed to model non-Markovian dynamics. These include hierarchical equations of motion (HEOM) [75], time-convolutionless and Nakajima–Zwanzig master equations [76], stochastic Schrödinger equations with colored noise [77], and chain mapping techniques [78]. Each of these methods presents trade-offs between accuracy, computational complexity, and ease of implementation, with HEOM being numerically exact but computationally expensive for large systems, while stochastic methods offer flexibility but require ensemble averaging.

Among these, the *pseudomode formalism* offers a conceptually clear and numerically efficient framework for the simulation of non-Markovian effects arising from reservoirs characterized by Lorentzian or near-Lorentzian spectral densities [41,42]. The formalism effectively replaces the structured environment with one or more auxiliary damped harmonic oscillators (pseudomodes) coupled directly to the system. The resulting dynamics can then be simulated using standard Lindblad master equations, thereby leveraging well-established numerical solvers while capturing essential memory effects.

Consider a general open quantum system described by the system Hamiltonian $H_{S}$ interacting with a bosonic reservoir. The total Hamiltonian of the system plus bath is

(13) $$H_{\mathrm{tot}}=H_{S}+\sum_{k}\omega_{k}b_{k}^{†}b_{k}+\sum_{k}\left(g_{k}Lb_{k}^{†}+g_{k}^{*}L^{†}b_{k}\right),$$

where *L* is a system operator coupling to the bath modes with creation and annihilation operators $b_{k}^{†}$ and $b_{k}$ and coupling strengths $g_{k}$. The bath influence is encoded in the spectral density

(14) $$J\left(\omega \right)=\sum_{k}{\left|g_{k}\right|}^{2}\delta \left(\omega -\omega_{k}\right).$$

When the spectral density can be approximated by a Lorentzian function, as is common in microwave cavity systems [79] and nanophotonic environments [80],

(15) $$J\left(\omega \right)=\frac{1}{\pi }\frac{g^{2}\kappa }{{\left(\omega -\omega_{a}\right)}^{2}+\kappa^{2}},$$

the pseudomode formalism shows that the exact reduced dynamics of the system can be obtained by coupling it to a single damped harmonic oscillator, or pseudomode, with frequency $\omega_{a}$, coupling strength *g*, and damping rate κ [42]. The total Hamiltonian of the combined system and pseudomode is

(16) $$H_{pm}=H_{S}⊗I+I⊗\omega_{a}a^{†}a+g\left(L⊗a^{†}+L^{†}⊗a\right),$$

where *a* and $a^{†}$ are the annihilation and creation operators of the pseudomode. The evolution of the joint density operator ρ(t) is governed by the Markovian master equation

(17) $$\frac{d\rho }{dt}=-i\left[H_{pm},\rho \right]+\kappa \left(a\rho a^{†}-\frac{1}{2}\left\{a^{†}a,\rho \right\}\right).$$

This representation effectively maps a non-Markovian open system problem onto an extended Markovian one, enabling the use of Lindblad-form master equations to simulate memory effects without explicitly dealing with integrodifferential equations or memory kernels. The parameter κ determines the width of the Lorentzian spectral density and thus controls the bath correlation time: small κ corresponds to strong non-Markovianity with long bath memory (as observed in high-Q cavities [81]), while large κ recovers the Markovian limit with rapid environmental decoherence. Recent work has extended this approach to multiple pseudomodes for complex spectral densities [82] and fermionic environments [83], demonstrating its versatility in modeling modern quantum devices where environmental memory effects are significant [84].

To explore the role of environmental memory in entanglement generation, we present a preliminary study of non-Markovian dynamics using the pseudomode formalism. By comparing protocols P1 and P2, we examine how memory effects influence the maximum entanglement achieved—effects that are typically neglected under the Markovian assumption.

We consider a single bosonic reservoir characterized by a Lorentzian spectral density, a widely used model that captures finite environmental correlation times. The system–reservoir coupling is implemented via the operator $L=\sum_{i=1}^{N}\left(S_{i}^{z}+S_{i}^{x}\right)$, which accounts for both dephasing and dissipative processes in a zero-temperature bath. The system Hamiltonian $H_{S}$ is given by Equation (1).

We note that richer non-Markovian features may emerge in the long-time dynamics. However, these effects lie beyond the scope of the present analysis, which is focused on fast, on-demand entanglement generation.

All numerical simulations in this work were performed using the QuTiP library [85]. Equation (17) was solved via QuTiP’s Monte Carlo solver mcsolve, with the number of trajectories chosen to ensure convergence. To balance the accuracy and computational cost, we adopted an adaptive Fock basis and validated its precision by evaluating the bosonic commutator $\left[a,a^{†}\right]$, maintaining a numerical error below $10^{-4}$. Additionally, the convergence of all physical observables was confirmed through comparisons with larger basis sizes during preliminary tests.

**Figure 9:**
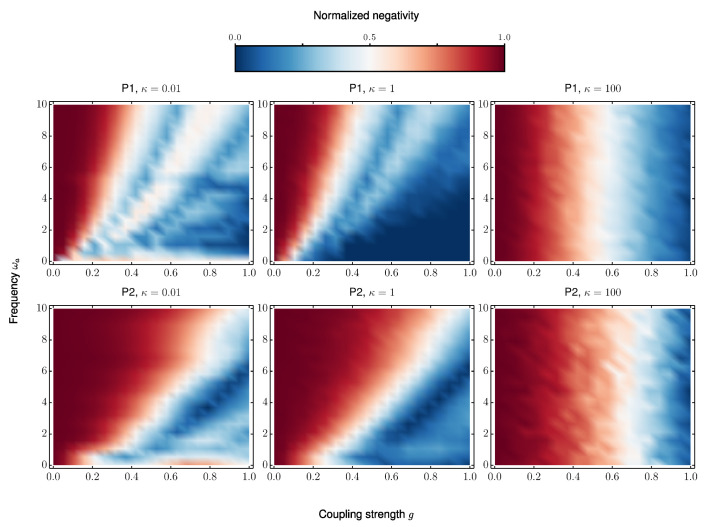
Maximum normalized end-to-end negativity achieved within an evolution time equal to twice the optimal transfer time of the closed system, for both protocols under non-Markovian dissipation modeled via the pseudomode method. Each column corresponds to a different spectral width κ of the Lorentzian reservoir: (left) κ=0.01 (strongly non-Markovian), (center) κ=1 (intermediate memory), and (right) κ=100 (Markovian limit). The top row displays results for P1 and the bottom row for P2. For small *g* and κ, P2 sustains high entanglement over a broader parameter region compared to P1. As *g* increases, both protocols deteriorate, but P2 remains more resilient. In the Markovian limit, both recover the Lindblad results, validating the approach.

Figure 9 presents heatmaps of the maximum normalized end-to-end negativity achieved within an evolution time equal to twice the optimal entanglement generation time of the corresponding closed system, as a function of the system–reservoir coupling strength *g* and the reservoir central frequency $\omega_{a}$. The three columns correspond to different values of the reservoir linewidth κ, which characterizes the spectral density of the environment and controls the degree of memory effects. The selected values—κ=0.01, κ=1, and κ=100—represent, respectively, a strongly non-Markovian regime with long bath memory, an intermediate regime where the bath correlation times are comparable to the system dynamics, and the Markovian limit.

For κ=0.01 and weak coupling (g<0.2), P1 can only reach high entanglement when the bath frequency $\omega_{a}$ is significantly detuned from the system’s eigenfrequencies. In contrast, P2 maintains high entanglement over a broader range of $\left(g,\omega_{a}\right)$ values, demonstrating greater robustness to resonance-induced decoherence. However, for larger coupling strengths (e.g., g≳0.4), both protocols fail to generate highly entangled states due to strong back-action from the environment.

When the bath linewidth is increased to κ=1, P1 exhibits similar behavior to the previous case, remaining highly sensitive to $\omega_{a}$ and only achieving strong entanglement in narrow regions of the parameter space. P2, on the other hand, shows an expanded region of high negativity, further confirming its resilience to moderate non-Markovian noise.

In the Markovian limit (κ=100), both protocols recover the results obtained using the Lindblad master equation in the previous section for g≲0.4, as expected. Notably, P2 continues to outperform P1 for stronger coupling values, retaining its advantage even as the system–reservoir interaction increases. These results highlight the superior robustness of P2 in the presence of both Markovian and non-Markovian environmental noise.

We emphasize that this is a preliminary analysis based on a simple yet physically relevant Lorentzian spectral density. More complex environments—such as sub-ohmic, super-ohmic, or multi-peaked spectra—would require additional pseudomodes to accurately capture their structures and may introduce qualitatively new dynamical features. A more detailed investigation of the full entanglement dynamics, beyond the short-time window associated with the maximum entanglement in the closed system, remains an important direction for future work.

## 4. Conclusions

We have conducted a comprehensive numerical study of two entanglement generation protocols in XX spin chains, evaluating their performance across spin magnitudes s=1/2, 1, and 3/2. Our analysis reveals that the dual-port architecture (P2) consistently achieves higher entanglement in shorter timescales than its staggered counterpart (P1) for all spin values considered.

In addition to its speed advantage, P2 demonstrates strong robustness against both diagonal and off-diagonal disorder, as well as local dephasing noise. This resilience is attributed to its design, which minimizes the excitation of the bulk spins through optimized boundary control and coupling symmetry, thereby enhancing the coherence and reducing the vulnerability to noise. We also extended our analysis to incorporate non-Markovian environmental effects using the pseudomode formalism. This approach allowed us to assess how environmental memory, arising from structured reservoirs with finite correlation times, affects the entanglement dynamics of both protocols. Our results show that P2 remains more robust than P1 across a wide range of spectral parameters, further confirming its suitability for implementation in noisy intermediate-scale quantum devices. These findings open up promising directions for future studies of long-time dynamics and more complex environments involving multiple pseudomodes and structured spectral densities. These features make P2 not only more efficient but also more scalable and robust, establishing it as a strong candidate for practical implementations in entanglement-based quantum information processing. Future work may explore the extension of this protocol to larger spin networks, the incorporation of more general non-Markovian effects [86], and embedding it within hybrid quantum architectures [87]. These directions could be further enriched by integrating advanced techniques such as color-engineered communication channels [88], star-like entanglement hubs for multi-qubit interfacing [89], and dissipative stabilization mechanisms for steady-state entanglement [90].

The XX spin chain with boundary magnetic fields studied in this work can be robustly implemented using several state-of-the-art quantum simulation platforms. In circuit QED architectures [79], nearest-neighbor XX interactions arise naturally between superconducting qubits (either flux or transmon types) dispersively coupled to a shared microwave resonator, while boundary *Z*-fields can be precisely engineered via local flux bias lines or microwave drives. Trapped-ion quantum simulators [91,92] offer an equally powerful alternative: phonon-mediated interactions, induced by laser fields, generate effective XX couplings, and site-resolved *Z*-fields can be realized through differential AC Stark shifts or magnetic field gradients.

More generally, our model naturally emerges in systems of *N* qubits dispersively coupled to a common bosonic mode via Jaynes–Cummings-type interactions [93]. In such setups, the effective *Z*-field is set by the qubit detunings, while spin-exchange interactions are mediated by virtual excitations of the bosonic mode. Crucially, this architecture enables full control over both the strength and spatial profile of the spin–spin couplings, by tuning the individual qubit mode detunings and coupling constants. As a result, arbitrary patterns of exchange interactions—such as the position-dependent profiles used in our protocols—can be engineered with high precision.

These experimentally established platforms, each offering complementary strengths in terms of coherence, control, and scalability, provide realistic and versatile routes by which to test our predictions and implement the proposed entanglement generation schemes in near-term quantum devices.

## Figures and Tables

**Table 1:** Peak end-to-end negativity N and corresponding evolution time *t* for P1 (subscript 1) and P2 (subscript 2). Times are in units of the weak coupling δ; $B_{A}=B_{B}=B$ are the optimal boundary fields.

Spin	$N_{1}$	$t_{1}$	$N_{2}$	$t_{2}$	*B*
1/2	1	22.50δ	1	13δ	3.7
1	0.75	13.9δ	0.94	9.8δ	2.9
3/2	0.62	10.54δ	0.9	7.45δ	4.7

## Data Availability

The datasets generated during this research are available from the corresponding authors upon reasonable request.

## Appendix A. Effective Dynamics

To complement the numerical results discussed in the main text, we present here a concise derivation of effective descriptions in two relevant limiting regimes that support entanglement generation between distant spins-1/2: (i) the dispersive regime of P2 and (ii) the strong dimerization limit, where the bulk dynamics are well described by a trimer model involving only a few sites.

### Appendix A.1. Effective Dispersive Hamiltonian for Protocol 2

We consider an *N*-site spin-1/2 XX chain with alternating couplings, described by

(A1) $$H=\sum_{i=1}^{N-1}J_{i}\left(S_{i}^{x}S_{i+1}^{x}+S_{i}^{y}S_{i+1}^{y}\right)+\sum_{i=1}^{N}B_{i}S_{i}^{z},$$

where $J_{i}=\Delta$ (strong) or δ (weak), and $B_{i}=\omega /2$ is a uniform magnetic field. We consider emitter (*e*) and receiver (*r*) qubits coupled at the boundaries via

(A2) $$H_{\mathrm{er}}=\frac{\omega }{2}\left(S_{e}^{z}+S_{r}^{z}\right)+\lambda \left(S_{e}^{+}S_{1}^{-}+S_{r}^{+}S_{N}^{-}+H.c.\right),$$

leading to the full Hamiltonian $H_{S}=H+H_{\mathrm{er}}$, with all $J_{i}=\Delta$ and λ=δ for P2.

Applying the Jordan–Wigner transformation maps the spin system to non-interacting fermions. The resulting Hamiltonian in the single-excitation subspace becomes

(A3) $$H_{S}^{\prime }=\sum_{k=1}^{N}E_{k}f_{k}^{†}f_{k}+\sum_{k=1}^{N}{\bar{\lambda }}_{k}\left(c_{e}^{†}f_{k}+{\left(-1\right)}^{k-1}c_{r}^{†}f_{k}+H.c.\right),$$

where

(A4) $$E_{k}=\Omega +2\Delta \cos\left(\frac{k\pi }{N+1}\right),$$

(A5) $${\bar{\lambda }}_{k}=\lambda \sqrt{\frac{2}{N+1}}\sin\left(\frac{k\pi }{N+1}\right),$$

Transforming to the interaction picture concerning $H_{0}=\sum_{k}E_{k}f_{k}^{†}f_{k}+\frac{\omega }{2}\left(S_{e}^{z}+S_{r}^{z}\right)$ gives

(A6) $${\bar{H}}_{S}\left(t\right)=\sum_{k}{\bar{\lambda }}_{k}\left[\left(c_{e}^{†}f_{k}+{\left(-1\right)}^{k-1}c_{r}^{†}f_{k}\right)e^{i\zeta_{k}t}+H.c.\right],$$

with detuning $\zeta_{k}=\omega -E_{k}$.

In the dispersive regime ${\bar{\lambda }}_{k}/\zeta_{k}\ll 1$, second-order perturbation theory yields an effective Hamiltonian that directly couples the boundary qubits via virtual excitations:

(A7) $$H_{\mathrm{eff}}=\chi \left(c_{e}^{†}c_{r}+c_{e}c_{r}^{†}\right),$$

with

(A8) $$\chi =\sum_{k}\frac{{\bar{\lambda }}_{k}^{2}}{\zeta_{k}}.$$

This mediated interaction allows coherent entanglement generation without significant excitation of the channel. The average number of excitations in the chain during evolution up to the entanglement generation time τ=π/4χ is

(A9) $$\langle n\rangle =\sum_{k}\int_{0}^{\tau }\frac{\langle f_{k}^{†}\left(t\right)f_{k}\left(t\right)\rangle }{\tau }dt\approx N{\left(\frac{\pi \delta }{2\Delta }\right)}^{2}.$$

Thus, the excitation leakage remains negligible as long as $\sqrt{N}\delta /\Delta \ll 1$.

Moreover, the third-order corrections scale as $N{\bar{\lambda }}_{k}^{3}/\zeta_{k}^{2}$, so the validity of the effective model requires

(A10) $$N\ll \frac{\zeta_{k}}{{\bar{\lambda }}_{k}}.$$

### Appendix A.2. Trimer Approximation in the Strong Dimerization Regime

In the opposite limit of static spin chains with alternating strong and weak couplings, the 7-site ABC spin chain can be reduced to an effective three-site (trimer) model involving only the sites labeled *A*, *B*, and *C*. For δ/Δ≪1, the chain effectively breaks into dimers weakly coupled via δ. In this regime, the dynamics relevant to entanglement generation can be captured by an effective trimer model involving only the boundary spins *A*, *C* and the central site *B*.

The effective Hamiltonian projected to the single-excitation subspace is

(A11) $$H_{\mathrm{trimer}}=\left(\begin{array}{ccc} 0 & \eta & 0\\ \eta & 0 & \eta \\ 0 & \eta & 0 \end{array}\right),$$

with effective coupling

(A12) $$\eta =\frac{\Delta }{2}\sqrt{1+3{\left(\frac{\delta }{\Delta }\right)}^{2}-\sqrt{1+6{\left(\frac{\delta }{\Delta }\right)}^{2}+{\left(\frac{\delta }{\Delta }\right)}^{4}}}.$$

This trimer mediates coherent oscillations between *A* and *C* through *B*, enabling the generation of high-fidelity entanglement. The first entanglement peak appears approximately at

(A13) $$t_{E}\approx \frac{t_{F}}{2}=\frac{\pi }{2\sqrt{2}\eta },$$

with the revival of the initial state at $t_{F}=\pi /\sqrt{2}\eta$.

This approximation generalizes to longer chains by symmetrically adding dimer pairs around the central site *B*, preserving low-energy trimer-like dynamics. However, the effective coupling η decreases exponentially with the chain length, leading to a corresponding increase in the entanglement timescale.

Both protocols allow for controlled entanglement generation between distant qubits by exploiting virtual excitation pathways. The effective Hamiltonians derived here enable fast, robust entanglement in the presence of weak system–bath interactions and minimal occupation of the intermediate chain.
